# Vulcanizing

**Published:** 1885-05

**Authors:** 


### VULCANIZING.

There are few dentists who fully realize what a tremendous engine of destruction they keep in their laboratories, or what serious peril they may have frequently encountered in working it. When one reflects upon the power of confined steam, and then remembers that very few dentists are careful to keep safety-valves in order, he wonders that every day does not bring its terrible tale of accidents from exploded vulcanizers. Here is a table of the expansive force of steam at certain temperatures, the result of experiments by members of the French Academy.

| Degrees of Temperature, Fahrenheit. | Elastic Force in lbs. per square inch. | Degrees of Temperature, Fahrenheit. | Elastic Force in lbs. per square inch. | Degrees of Temperature, Fahrenheit. | Elastic Force in lbs. per square inch. | Degrees of Temperature, Fahrenheit. | Elastic Force in lbs. per square inch. |
|---|---|---|---|---|---|---|---|
| 212. | 14.7 | 300.28 | 66.12 | 341.78 | 117.6 | 392.46 | 220.5 |
| 233.96 | 22.05 | 307.05 | 73.05 | 350.78 | 132.3 | 398.48 | 235.2 |
| 250.52 | 29.4 | 314.24 | 80.85 | 358.88 | 147. | 403.82 | 249.0 |
| 263.84 | 36.7 | 320.36 | 88.2 | 366 85 | 161.7 | 408.92 | 264.6 |
| 275.18 | 44.15 | 326.26 | 95.55 | 374.00 | 176.4 | 413.78 | 279.3 |
| 285.08 | 51.45 | 331.70 | 102.9 | 380.66 | 191.1 | 418.46 | 294. |
| 293.72 | 58.8 | 336.94 | 110.85 | 382.94 | 205.8 | | |

Many dentists or assistants run a vulcanizer almost by guess. The thermometer is frequently cracked, and so does not register accurately. The flame is unsteady, the heat is constantly changing, and it is not uncommon to see the mercury rising to the neighborhood of 400°, through carelessness and recklessness. Not only does such heedless rashness endanger human life, but it seriously damages the products of vulcanization. A piece of work subjected to such extreme temperatures cannot be as perfect as one that is properly vulcanized, and no skilled workman who cares for uniformity of results will be so unscientific in his methods.

There is probably less vulcanizing done in our laboratory than in that of most dentists, but for fifteen years we have not allowed a piece to be done unless there was attached to the vulcanizer a reliable gas regulator for maintaining the heat at a constant and unvarying point. For ten years we have had in use one devised by Prof. Coolidge, of Boston, and manufactured by the Buffalo Dental Manufacturing Company, and never during this time has the thermometer varied three degrees from the maximum point, in vulcanizing. Even were the thermometer destroyed, or out of order, it would not interfere with perfect vulcanization. To say nothing of the better products of such a machine, or the complete safety consequent upon its use, it will save its cost in a short time by its convenience, for it is automatic in its action and requires no watching, putting itself out at the expiration of the time for which it is set. Where there is a gas supply, it seems almost criminal recklessness to attempt vulcanization without some kind of regulator, now that they are made so perfectly efficient.

